# Optimized AAV rh.10 Vectors That Partially Evade Neutralizing Antibodies during Hepatic Gene Transfer

**DOI:** 10.3389/fphar.2017.00441

**Published:** 2017-07-17

**Authors:** Ruchita Selot, Sathyathithan Arumugam, Bertin Mary, Sabna Cheemadan, Giridhara R. Jayandharan

**Affiliations:** ^1^Department of Biological Sciences and Bioengineering, Indian Institute of Technology Kanpur, India; ^2^Department of Hematology and Centre for Stem Cell Research (CSCR), Christian Medical College Vellore, India

**Keywords:** AAV, gene therapy, immune-escape, neutralizing antibody, T-cell, B-cell, confocal microscopy

## Abstract

Of the 12 common serotypes used for gene delivery applications, Adeno-associated virus (AAV)rh.10 serotype has shown sustained hepatic transduction and has the lowest seropositivity in humans. We have evaluated if further modifications to AAVrh.10 at its phosphodegron like regions or predicted immunogenic epitopes could improve its hepatic gene transfer and immune evasion potential. Mutant AAVrh.10 vectors were generated by site directed mutagenesis of the predicted targets. These mutant vectors were first tested for their transduction efficiency in HeLa and HEK293T cells. The optimal vector was further evaluated for their cellular uptake, entry, and intracellular trafficking by quantitative PCR and time-lapse confocal microscopy. To evaluate their potential during hepatic gene therapy, C57BL/6 mice were administered with wild-type or optimal mutant AAVrh.10 and the luciferase transgene expression was documented by serial bioluminescence imaging at 14, 30, 45, and 72 days post-gene transfer. Their hepatic transduction was further verified by a quantitative PCR analysis of AAV copy number in the liver tissue. The optimal AAVrh.10 vector was further evaluated for their immune escape potential, in animals pre-immunized with human intravenous immunoglobulin. Our results demonstrate that a modified AAVrh.10 S671A vector had enhanced cellular entry (3.6 fold), migrate rapidly to the perinuclear region (1 vs. >2 h for wild type vectors) *in vitro*, which further translates to modest increase in hepatic gene transfer efficiency *in vivo*. More importantly, the mutant AAVrh.10 vector was able to partially evade neutralizing antibodies (~27–64 fold) in pre-immunized animals. The development of an AAV vector system that can escape the circulating neutralizing antibodies in the host will substantially widen the scope of gene therapy applications in humans.

## Introduction

Gene therapy using recombinant Adeno-associated virus (Serotypes AAV1 to AAV12) has gained significant attention after early success with several pre-clinical and clinical studies (De et al., 2006; Zincarelli et al., 2008; Mendell et al., 2010; Mingozzi and High, 2011; Nathwani et al., 2011). However, for this strategy to be routinely applicable in a larger number of patients, it is essential to mitigate the immune manifestations of AAV vectors in the host. Innate immune response profile during AAV infection in the host, is well-characterized in terms of molecular regulators/pathways (Mays and Wilson, 2010; Jayandharan et al., 2011; Balakrishnan et al., 2013; Hareendran et al., 2013; Sen et al., 2014) and is known to lead to adaptive immune response by memory T cell reactivation (Selot et al., 2014). In the clinical trial for hemophilia B, hepatic gene delivery with AAV2 vectors achieved transient (~6 weeks) yet therapeutic expression of coagulation factor IX (FIX) but subsequent CD8+ T cell response destroyed AAV infected hepatocytes (Manno et al., 2006; Mingozzi et al., 2007). A similar immunogenic response to AAV is evident from other clinical trials as well (Mcphee et al., 2006; Brantly et al., 2009). These data suggest that any long term AAV vector transgene expression will require approaches to minimize capsid specific cellular immune responses. This will need AAV vectors that express therapeutic levels of the transgene at low vector doses to circumvent dose dependent immuno-toxicity (Manno et al., 2006; Mingozzi et al., 2007).

AAV vectors also have a broad host-range (Gao et al., 2002; Bossis and Chiorini, 2003). Thus, their natural exposure in humans results in AAV specific neutralizing antibodies (Nab). Their sero-positivity in humans range between 20~70%, with the lowest and highest seroprevalance seen for AAVrh.10 and AAV2, respectively (Boutin et al., 2010; Thwaite et al., 2014). Some of these Nab are also known to be cross reactive to multiple AAV serotypes (Calcedo et al., 2009). These data underscore the need to have vectors which not only can bypass the *de novo* cellular immune response but also capable of evading pre-existing humoral immunity, for them to be universally applicable.

To overcome such immunological roadblocks during AAV mediated gene transfer, it is crucial to comprehend the host-virus biology and utilize such information to develop optimal gene transfer strategies. Various studies have shown that cellular entry and ubiquitination of the AAV capsid are major rate-limiting steps, which also increases its antigen presentation and leads to cellular or humoral immune response (Finn et al., 2010; Karman et al., 2012). Since systemic administration of proteasomal inhibitors may not be feasible in humans (Rajkumar et al., 2005), we and others have demonstrated that modification of the capsid amino acids that are the targets for phosphorylation and ubiquitination can be a feasible option to improve AAV mediated gene expression (Gabriel et al., 2013; Mingozzi et al., 2013a). We have evaluated AAV1, 2, 5, and 8 vectors with capsids altered at phosphodegron like regions, which are specific targets of phosphorylation/ubiquitination and demonstrated improved gene transfer efficiency (Sen et al., 2013a). In search of an immunologically naïve AAV vector, we have investigated if modifications of AAVrh.10 serotype in phosphodegron-like-regions are beneficial. AAVrh.10 was derived from rhesus macaques and belongs to Clade E (Gao et al., 2004). Comparison of AAV serotypes 1 through 9 and rh.10 in neonatal mice has demonstrated superior transgene expression for AAVrh.10 vectors up to 100 days of investigation (Hu et al., 2010). In preclinical models, AAVrh.10 vectors have been highly efficient in delivering coagulation factor (F) VIII at either lower doses (FVIII levels ~5% at 2 × 10^12^ vg/kg) or higher doses (FVIII levels ~19% at 7 × 10^12^ vg/kg) without any evidence of immuno-toxicity until ~22 months after gene transfer (Hu and Lipshutz, 2012). AAVrh.10 vectors are also known to have lower cross reactivity to Nabs. In a comparative study that assessed non-human primate derived AAV such as AAV 7, 8, and rh.10 to deliver alpha-1 anti-trypsin gene, it was found that AAV rh.10 greatly improves the transgene expression by ~300% in mice with antibodies to AAV (De et al., 2006). We have now further improved the utility of this promising vector system by targeting phosphodegron like regions in AAVrh.10, which improved their cellular uptake and intracellular trafficking, enhanced their gene expression and demonstrated considerable resistance to neutralization by human intravenous immunoglobulin (IVIG).

## Methods

### Animals

We used C57BL/6J mice in this study. The study was performed according to Institutional Guidelines for Animal Care at Indian Institute of Technology, Kanpur, India and Christian Medical College, Vellore, India. The protocol was approved by the Institutional Animal Ethics Committee and Institutional Biosafety Committee at the Christian Medical College, Vellore and Indian Institute of Technology, Kanpur, India.

### Cell lines

Human cervical carcinoma cell line (HeLa) and human embryonic kidney cell line (HEK293T) cell lines were purchased from the American Type Culture Collection (ATCC, Rockville, MD, USA). AAV293 cells were obtained from Stratagene (La Jolla, CA, USA).

### Mutagenesis of AAVrh.10 capsid

Homologous capsid residues in and around the phosphodegrons that were effective in AAV2 were chosen for mutagenesis in AAVrh.10 capsid. The lysine residues were chosen based on their probability of ubiquitination (UBIPRED, http://iclab.life.nctu.edu.tw/ubipred/). Mutations (S or T→A and K→R) were introduced on the capsid region of AAVrh.10 plasmid by a commercial mutagenesis kit (QuikChange II XL, Agilent technologies). The list of mutations and the primer sequences used for mutagenesis are listed in Supporting Information Table S1.

### Generation of recombinant AAV vectors

Self-complementary AAVrh.10 WT or their mutants expressing luciferase was generated as published earlier (Gabriel et al., 2013; Sen et al., 2013a). The titer of vectors were measured by three independent slot-blot analysis and the mean titer value was denoted as viral genomes (vgs)/ml (Kube et al., 1997; Sen et al., 2013a).

### *In vitro* transduction assay

The efficiency of the mutant vectors was first assessed in HeLa or HEK293T cells. Cells were infected with 2 × 10^3^ vgs/cell of either WT-AAVrh.10 or the mutant vectors. Forty-eight hours post-transduction, luciferase activity was estimated using Luciferase Reporter Assay Kit (Biovision, CA, USA) in a GlowMax 20/20 luminometer (Promega, WI, USA). Mean of percentage relative luminescence unit (RLU) positivity from replicate samples and data generated from three independent experiments were used for comparison. In addition, the levels of neutralization antibodies produced in animals administered with WT-AAVrh.10 or the mutant vectors was performed by a neutralization assay described earlier (Calcedo et al., 2009).

### *In vivo* studies

To evaluate the hepatic transduction potential of the modified vectors, 8–12 weeks-old C57BL/6 mice were administered intravenously with 5 × 10^10^ vgs each of WT-AAVrh.10 or the mutant vector containing firefly luciferase gene. The gene expression was measured by serial bioluminescence imaging (IVIS Spect-CT, Caliper Life Sciences, Hopkinton, MA).

### Estimation of vector copy numbers

To estimate the cellular uptake of the viruses, HeLa cells were infected with 2 × 10^3^ vgs of WT-AAVrh.10 or the mutant S671A vectors. Forty-eight hours later, we isolated the DNA by a commercial kit (Qiagen, Valencia, CA). Similarly, to determine the transduction efficiency of AAV vectors *in vivo*, we isolated DNA from liver tissue of mice injected with WT-AAVrh.10 or the mutant S671A vectors, 72 days after vector administration. Vector genome copy numbers per diploid human/mouse genome were determined by TaqMan probes and primers designed against the AAV inverted terminal repeat (ITR) sequence or bovine growth hormone polyadenylation signal in an ABI Prism 7500 Sequence Detection System (Life Technologies) as described previously (Aurnhammer et al., 2012; Sen et al., 2013a,b; Wang et al., 2013; Lock et al., 2014).

### Study of intracellular trafficking of AAV by time-lapse confocal microscopy

Carbocyanine (Cy3) fluorescent dye (GE Healthcare, Buckinghamshire, United Kingdom) labeled AAV were prepared as described previously (Bartlett et al., 2000). HeLa cells were then infected with these labeled AAV particles at an MOI of 1 × 10^5^. We then localized the labeled AAV particles by confocal laser scanning microscopy at an excitation wavelength of 552 nm (Olympus Flouview1200 Confocal Microscope, Japan). Images were processed by Olympus Fluoview FV1000 confocal software. To further analyze the rate of nuclear translocation of the wild type and mutant vectors, images from mock and AAV infected cells were studied by ImageJ analysis software (NIH, Bethesda, MD, USA).

A time-lapse movie was also obtained with images captured every 5 min. After 12 h of live cell imaging, cells were fixed with 4% para-formaldehyde and the nuclei stained with Hoechst dye (33258) (Sigma Aldrich, St. Louis, USA). Images were captured in an Olympus Fluoview 1200 confocal microscope.

### Evaluation of Nab-escape potential of AAVrh.10 vectors

C57BL/6 mice (8–12 weeks-old) were administered with 2 mg of human IVIG intra-peritoneally. Twenty-four hours later, AAVrh.10, WT or S671A vectors encoding luciferase were administered at a dose of 2 × 10^10^ vgs *via* the tail vein. The comparison group received only AAV vectors. The experiment was repeated independently, with IVIG sourced from two different commercial vendors (Intratect®, Biotest, Dreieich, Germany and IVIGlob® EX, VHB Life Sciences Ltd., Mumbai, India). To measure the transduction efficiency in the presence and absence of IVIG, small animal imaging was performed for all the animals under investigation.

### Statistical analysis

Comparison between the test and control groups was done by analysis of variance (ANOVA) or Student *t*-tests, and a *p-value* < 0.05 is denoted as statistically significant.

## Results

### AAVrh.10 mutants have improved transduction *in vitro*

**Figure 1:**
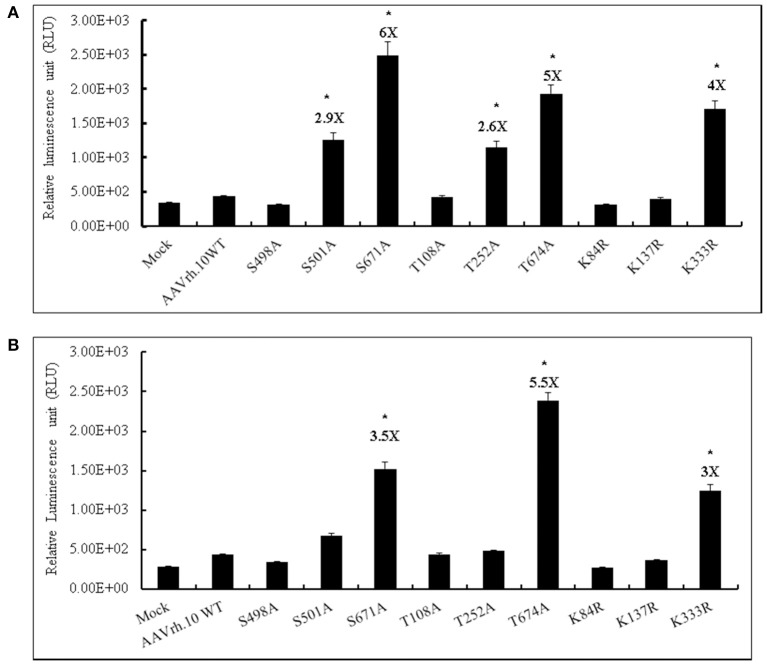
Transduction efficiency of AAVrh.10 mutants *in vitro*. **(A)** HeLa or **(B)** HEK293T cells were either PBS-treated or transduced with 2 × 10^3^ vgs/cell of AAVrh.10-WT or AAVrh.10- mutant vectors. Forty-eight hours later, cells were lysed and the luciferase expression was measured by a Luciferase Reporter Assay kit (Biovision, CA, USA). The data are from three independent experiments. ^*^p < 0.05, when compared to the WT- infected cells.

We have generated a total of nine mutant AAVrh.10 vectors by targeting particular S, T, K amino acids on the capsid that are homologous to the phosphodegrons reported in AAV2 capsid. We then evaluated these mutant vectors (MOI, 2 × 10^3^vgs/cell) for their transgene expression in HeLa and HEK293T cells. The data in Figure 1, shows that the AAVrh.10 mutant vectors S671A, T674A, and K333R had a significantly higher luciferase expression in HeLa (4–6 fold) (Figure 1A) or 293T cells (3–5.5 fold) (Figure 1B) when compared to WT-AAVrh.10 infected cells. The highest transdcution was seen with the AAVrh.10-S671A vector in both the cell types tested (6 fold vs. WT-AAVrh.10 in Hela and 3.5 fold vs. WT-AAVrh.10 in HEK293T cells).

### Modified AAVrh.10 vectors have improved cellular entry and translocate rapidly into the nuclear compartment

**Figure 2:**
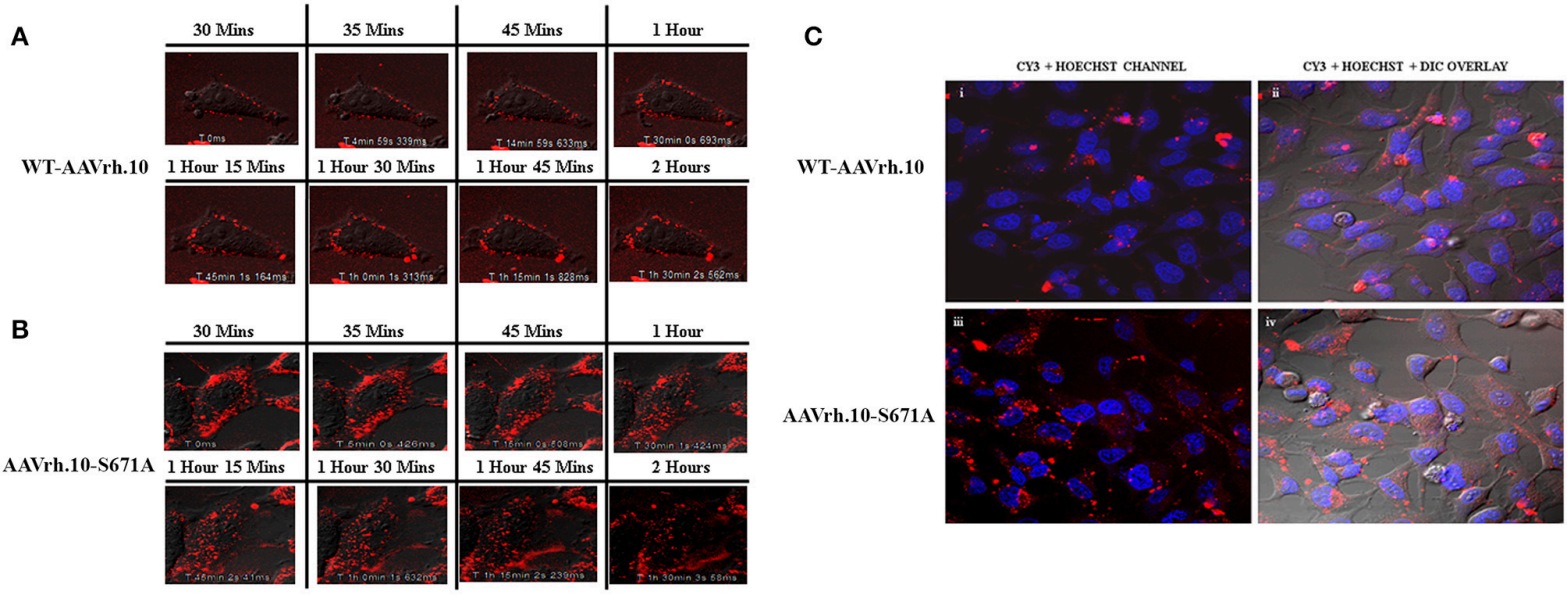
AAVrh.10 mutant vectors demonstrate increased rate of intracellular trafficking *in vitro*. HeLa cells were infected with Cy3 fluorescent dye labeled AAVrh.10 vectors at an MOI of 1 × 10^5^ vgs. Images were obtained at different timepoints in an Olympus confocal microscope. Live cell images of **(A)** WT-AAVrh.10 and **(B)** AAVrh.10-S671A vectors were captured serially for 2.5 h. Twelve hours post live imaging, the cells were fixed with 4% para-formaldehyde. Cells were then observed after staining the nucleus with Hoecsht dye, and images were captured under an Olympus confocal microscope **(C-i,ii)** representative images of fixed cells infected with WT-AAVrh.10 and AAVrh.10-S671A are shown in the Cy3+Hoechst channel and **(C-iii,iv)** Cy3+Hoechst+ differential interference contrast (DIC) channel.

Since the mutant AAVrh.10 vectors had enhanced transduction, we then evaluated the basis for this observation. As a first step, we determined the rate of cellular entry/uptake of the best mutant AAVrh.10-S671A in comparison to WT-AAVrh.10 vectors in HeLa cells. Three hours post-infection, a cellular entry assay by quantitative PCR analysis was performed. Significantly, our data shows a ~3.6 fold higher vector genome copy number per diploid genome in AAVrh.10-S671A infected cells in comparison to WT-AAVrh.10 vector infected cells (Supporting Information Figure S1). To further determine their rate of intracellular trafficking, we fluorescently labeled the best mutant, AAVrh.10-S671A with a Cy3 dye and visualized the movement of labeled virions from the cell membrane to the nucleus by live-cell imaging. Our analysis showed a clear difference in dynamics of intracellular trafficking of the AAVrh.10 mutant when compared to WT vectors (Supporting Information Video 1 for WT-AAVrh.10 and Supporting Information Video 2 for AAVrh.10-S671A). The images captured at various time points after infection (Figure 2) show that the mutant vector AAVrh.10-S671A enter HeLa cells efficiently and demonstrate a rapid accumulation within the cell. For example, as early as 1 h post-infection, AAVrh.10-S671A vectors rapidly translocate into the peri-nuclear space as opposed to the WT-AAVrh.10 vector, which are localized either to the cell membrane or in the process of being internalized into the cell (Figures 2A,B). Furthermore, quantitation of these viral particles, demonstrate that a greater proportion of mutant vectors (~6–30 fold vs. WT vectors) are localized in the nuclear compartment within the first hour (Supporting Information Figure S2). These findings were further corroborated by imaging the fixed cells at ~12 h post-infection and the nuclei counter-stained with Hoechst stain. Figure 2C demonstrates a significantly higher accumulation of the Cy3 labeled mutant vectors within the nucleus in comparison to the WT vectors. To further understand, if this rapid trafficking is because of an altered ubiquitination profile of S671A vectors, we performed an *in vitro* ubiquitination and immunoblot assay. As shown in Supporting Information Figure S3, the S671A mutant had a ubiquitination profile similar to WT vector, suggesting that altered ubiquitination is not responsible for the improved cellular trafficking seen with these vectors.

Taken together, our data suggest that the mutant AAVrh.10-S671A vectors have better cellular uptake possibly due to reduced phosphorylation of the viral capsid and also traffic rapidly into the nucleus which further translates into an improved transgene expression. Our data also confirms that cellular entry and intracellular trafficking are major rate limiting steps in the transduction of WT-AAVrh.10 vectors as has been reported for other serotypes like AAV2 (Ding et al., 2005; Nonnenmacher and Weber, 2012; Xiao and Samulski, 2012; Liu et al., 2013).

### AAVrh.10-S671A vectors have modestly improved hepatic transgene expression *in vivo*

**Figure 3:**
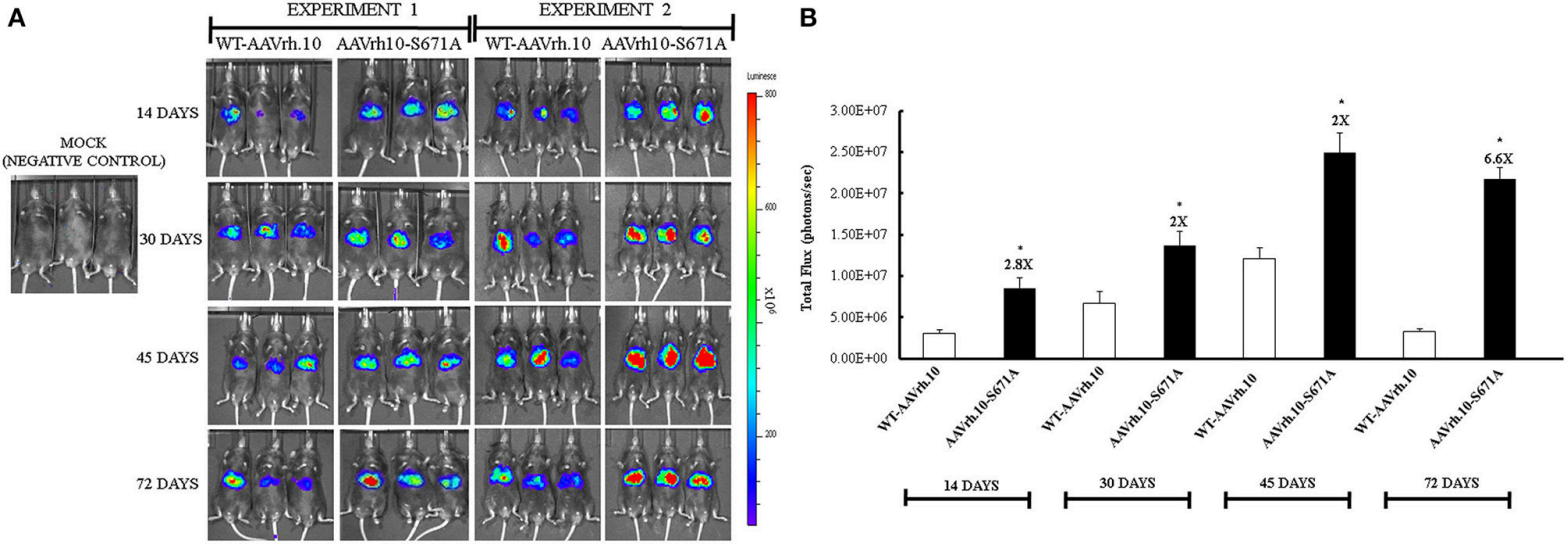
Transduction characteristics of AAVrh.10-S671A mutant vectors *in vivo*. **(A)** Luciferase activity was detected at different time points after gene transfer with 5 × 10^10^ vgs/animal of WT-AAVrh.10 or AAVrh.10-S671A vectors. Two independent sets of packaged vectors were used for the *in vivo* experiments 1 and 2. **(B)** Quantification of data from panel a. ^*^p < 0.05, when compared to the WT- administered mice. Representative images are shown in the figure.

To study the effect of mutant vectors *in vivo*, we administered mice with either 5 × 10^10^ vgs each of WT-AAVrh.10 or AAVrh.10-S671A mutant vectors. Luciferase gene expression was documented by small animal imaging at 14, 30, 45, and 72 days post-vector administration. In animals administered with the mutant vectors, a modest increase in luciferase expression was seen (Figures 3A,B). To further corroborate this data, viral genome copies were estimated in the liver tissue of all the mice by quantitative PCR. The AAVrh.10-S671A mutant vector had an × 3 fold higher copy number than the WT-AAVrh.10 (2.07 vs. 0.64, Table 1).

**Table 1:** Vector bio-distribution in the liver of C57BL/6 mice administered with WT-AAVrh.10 and its mutant vector 72 days post-hepatic gene transfer.

**Vector type**	**Vector copies per mouse diploid genome (mean ± *SD*)**
WT-AAVrh.10	0.64 ± 0.45
AAVrh.10-S671A	2.07 ± 0.73

*Values denote copy number of vectors in mouse diploid genome*.

### Capsid modified AAVrh.10-S671A vectors evade neutralizing antibodies *in vivo*

To test if mutant vectors are able to escape neutralization antibodies *in vivo*, AAVrh.10-S671A mutant and WT vectors were administered into C57BL/6 mice that had been pre-treated with IVIG (2 mg/animal). This dose of IVIG was chosen based on IVIG titration experiments and dose finding studies reported previously (Lin et al., 2008; Gabriel et al., 2015).

**Figure 4:**
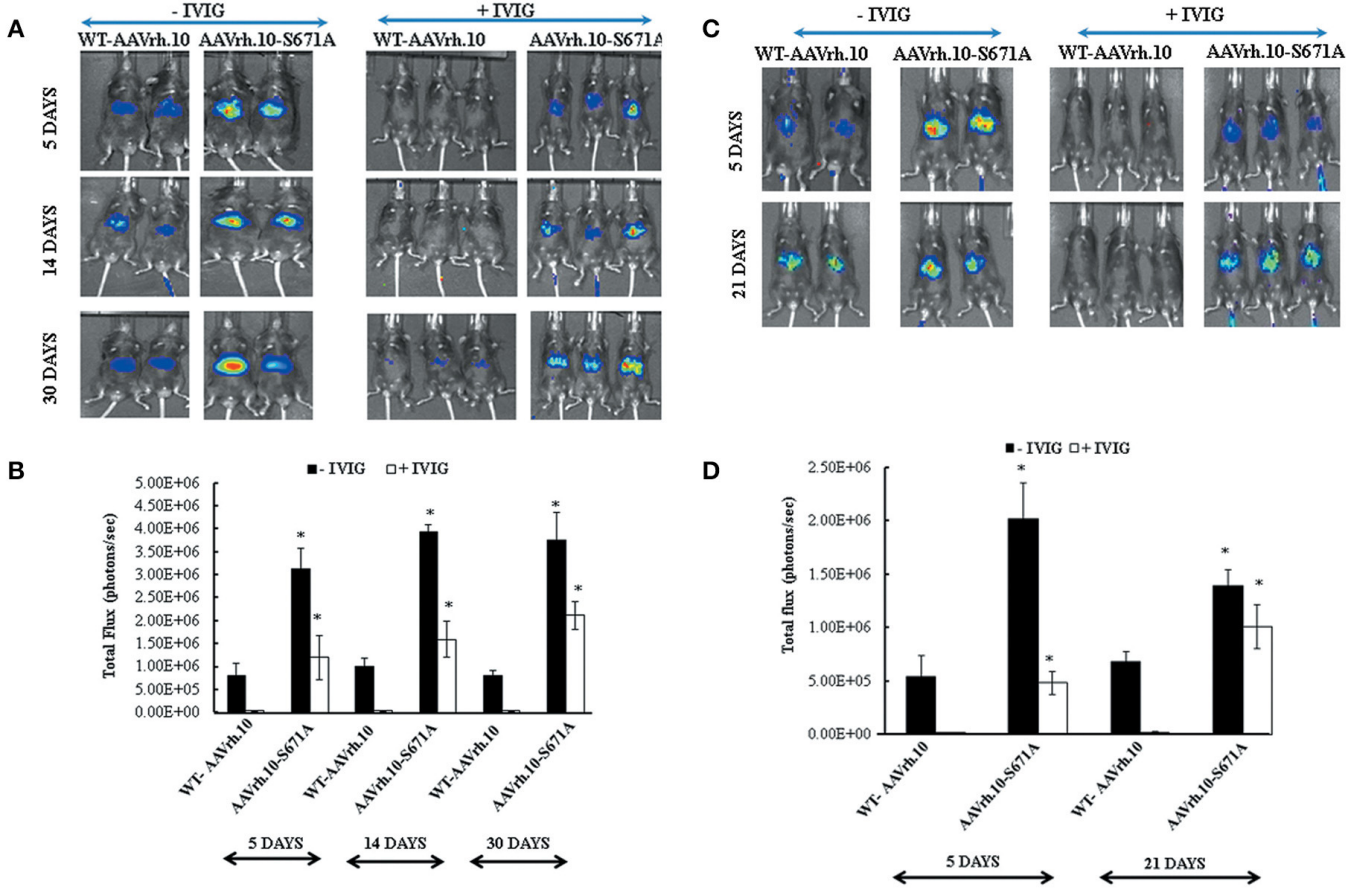
AAVrh.10-S671A mutant vectors resist neutralization by circulating antibodies *in vivo*. IVIG from Intratect®, Biotest, Dreieich, Germany (2mg/animal) was injected intra-peritoneally into C57BL/6 mice 24 h prior to administration of the WT-AAVrh.10 and the AAVrh.10-S671A vectors. C57BL/6 mice that were not administered with IVIG were also injected with these vectors (control group). Panel **(A)** Bio-luminescence imaging of mice administered with WT-AAVrh.10 or its mutant in the presence or absence of IVIG at 5, 14, and 30 days post-vector transduction. Panel **(C)** represents the experiment performed under the same conditions but with a different IVIG source (IVIGlob® EX, VHB Life Sciences Ltd, Mumbai, India). Panel **(B,D)** represent quantified data of the luciferase expression obtained from **(A,C)**, respectively. ^*^p < 0.05, in comparison to control group. Representative images are shown in the figure.

In the present study, our neutralization experiments was carried out using IVIG from two different commercial vendors to study the pattern of reporter gene expression when challenged with pooled serum (antibodies) from donors of variable ethnicity. As can be seen in Figure 4, all the six animals that had received IVIG and were further injected with AAVrh.10-S671A vectors had relatively higher luciferase expression. In comparison, in animals that had received IVIG and WT-AAVrh.10 vectors, the luciferase expression was either negligible or abrogated. Further quantitative analysis revealed that the partial rescue in gene expression in mutant vector administered mice in the presence of IVIG was ~27 to 64 fold (Figures 4B,D). In our *in silico* prediction for T cell and B cell epitopes by IEDB server (Immune Epitope Data Base) (Ponomarenko et al., 2008), we identified a putative epitope (GMVWQNRDVY LQGPIWAKIP HTDGNFHPSP LMGGFGLKHP PPQILIKNTP VPADPPTTFS QAKLASFITQ YST) encompassing S671 residue in AAVrh.10 capsid. It is possible that this antibody recognition epitope is perturbed in the mutant AAVrh.10 S671A vector and confers partial resistance to circulating Nabs. However further detailed studies are required to understand the basis of this phenomenon. This data was further corroborated by the 2-fold reduction in cross neutralization of AAVrh.10 S671A vector in a neutralization antibody assay (Supporting Information Table S2).

## Discussion

The present study was designed to understand if modification of phosphodegron-like regions in an alternate serotype AAVrh.10 can improve (a) cellular uptake (b) intracellular trafficking (c) transgene expression and (d) minimize immune-recognition by circulating Nabs to facilitate gene transfer in a sero-positive setting.

AAVrh.10 vector is known to be suitable for hepatic gene therapy and for neonatal gene transfer (Zincarelli et al., 2008; Hu et al., 2010; Miyake et al., 2012). AAVrh.10 vectors have been used effectively in delivering lysosomal enzyme galactocerebrosidase (GALC) gene in a postnatal mouse model of Krabe's disease and mice that received gene therapy had an extended life span of upto 8 months (Rafi et al., 2012). AAVrh.10 vectors when compared with AAV7 and AAV8 or AAV9 were found to have sustained hepatic transgene expression (F.IX and EGFP) in C57BL/6 mice (Nathwani et al., 2009; Dane et al., 2013). In more recent studies involving AAVrh.10 vectors, the efficiency of this serotype for gene transfer into brain has been proven (Swain et al., 2014; Vincent et al., 2014). AAVrh.10 vectors also have been used in re-dosing studies, where they restored transgene expression in adult mice administered with AAV8 vectors in the neonatal stage (Wang et al., 2012). This effect is possibly due to the lack of humoral immune cross-reactivity between AAV8 and AAVrh.10 serotypes (Nathwani et al., 2009). Although neonatal gene transfer using AAVrh.10 offers several advantages including, a higher vector: cell ratio permitting their administration at low doses, an ability to target stem and progenitor cells, the immune response directed against the virus after i.v. administration into adult animals is not well-understood (Waddington et al., 2004). In anticipation of testing these modified vectors in adult therapeutic models of disease like hemophilia, we decided to modify putative epitopic regions in AAVrh.10 vectors.

Our initial studies were focused on deducing the effect of viral capsid modifications on the cellular uptake and trafficking process. To the best of our knowledge, the present study is the first to have studied this phenomenon for AAVrh.10 serotype. Indeed, our data shows that the AAVrh.10-S671A vector had rapid cellular entry and trafficking profile in the cellular cytoplasm, and localized to the perinuclear region within the first 2 h after infection. Similar data was observed when we analyzed the trafficking of other alternate serotypes such as AAV1 (WT and S669A) *in vitro* (Supporting Information Figure S4). These findings also corroborate earlier reports where only a fraction (~27%) of WT-AAV2 vectors was internalized into the nucleus within the first 2 h after infection (Xiao and Samulski, 2012). One of the plausible mechanisms for this phenomenon seen across different AAV serotypes is that the removal of phosphorylation moieties in the mutants allows a greater proportion of intact virions enter the cell possibly by improved receptor binding. This may further expedite their endosomal processing and escape and facilitate their rapid translocation into the nuclear compartment. Nonetheless, further detailed studies are required to understand the basis of this phenomenon. We next wished to examine if the rapid intra-cellular trafficking of these modified AAVrh.10 could also translate into enhanced gene expression. This is important since increased vector transduction does not always lead to a benefit in gene expression (Lisowski et al., 2013). Many of the mutants of AAVrh.10 showed increased transduction capability *in vitro* in two different human cell lines, HeLa and 293T. *In vivo*, the AAVrh.10-S671A mutant transduced murine hepatocytes with modest efficiency when compared to the WT-AAVrh.10 vectors. Thus, it is possible that the increased gene expression that is seen for these mutant vectors can be partially attributed to their improved cellular uptake and/or subsequent enhancement in intracellular trafficking. It must be noted that this mutation AAVrh.10-S671A is a serine→alanine mutation homologous to the site S668A in AAV2 vector, that had showed a similar increase in transgene expression *in vivo* in our previous studies (Gabriel et al., 2013).

In the clinical trial conducted in patients with severe hemophilia B, the high titer of Nab (1:17) noted in one of the patient who received 2 × 10^12^ vg/kg of AAV2-hFIX vectors in the high dose group resulted in reduced FIX levels (Manno et al., 2006). Similarly, in primate models, Nab titers >1:10 dramatically impaired hepatic transduction of AAV8 vectors. The formation of memory B cells and T cells due to natural exposure to AAV in humans is an additional factor that compromises secondary administration of AAV vectors (Mingozzi and High, 2011). These data underscore the need to investigate vector/serotype specific cellular and humoral response in patients prior to gene therapy. Approaches such as transient immuno-suppression by use of clinically approved immunosuppressive drugs during gene transfer (Mingozzi et al., 2012, 2013b) or plasmapheresis (Monteilhet et al., 2011) can be beneficial, but reactivation and clonal expansion of AAV specific memory B cells in such individuals have to be evaluated.

Alternatively, the possibility of modification of the molecular structure of vector and disruption of immunogenic domains has helped in designing virions that escape neutralization by AAV specific antibodies. Owing to the fact that multiple modifications can potentially destabilize the AAV capsid, it is pertinent to choose the specific amino acid targets and optimize best combinations so that the desirable properties such as immune-escape, enhanced packaging ability, infectivity, and restricted tissue tropism are achieved (Maersch et al., 2010). Error prone PCR and DNA shuffling methods have been employed to identify Nab escape AAV (Maheshri et al., 2006; Perabo et al., 2006). In addition an immune stealth phenotype has been achieved by shuffling antigenic domains of multiple serotypes of AAV (Grimm et al., 2008; Koerber et al., 2008; Li et al., 2008). In our previous studies, we have generated phosphodegron modified AAV2 vectors with better transduction efficiency and a reduced cross neutralization by Nabs (Gabriel et al., 2013). Based on this, we reasoned that similar modifications in alternate serotypes such as AAVrh.10 could generate vectors with an immune escape potential. Indeed, our data shows that modified AAVrh.10 (S671A) mutants when tested in the presence of human IVIG rescued hepatic gene expression. This S671A modification however resulted only in a partial rescue of transgene expression suggesting that additional epitopes need to be identified. Further detailed investigations are required to understand the basis of resistance to the circulating Nabs by the modified AAVrh.10 vectors. Furthermore, their performance in a higher animal model (canine or primates) closer to human setting needs to be evaluated to understand any off target effects, before their potential application in a therapeutic setting.

Our data demonstrates that it is possible to improve the immune escape potential of AAVrh.10 vectors by selective modifications to the viral capsid. Such vectors in combination with other immunosuppressive regimens (Hareendran et al., 2016) offer the possibility of universal application of this promising vector system in human gene therapy.

## Author contributions

GJ conceptualized and designed the experiments. RS, SA, BM, and SC performed the experiments. RS and GJ analyzed and interpreted the data and wrote the paper. All authors discussed the results and commented on the manuscript.

### Conflict of interest statement

The authors declare that the research was conducted in the absence of any commercial or financial relationships that could be construed as a potential conflict of interest.

## Supplementary material

Supporting Information Figure S1 Cellular uptake is enhanced with modified AAVrh.10-S671A vectors. HeLa cells were infected at an MOI of 5 × 10^3^ with AAVrh.10-WT or S671A vectors. Genomic DNA was isolated 3 h post-infection and the vector copy number was measured by qPCR. The data was analyzed by student's *t*-test and the vector copy number in diploid genome are expressed as mean ± *SD* of two independent triplicate analysis. ^*^*p* < 0.05 when compared to AAVrh.10-WT infected cells.

Supporting Information Figure S2 Quantitative estimation of viral trafficking in the cytoplasmic **(A)** or nuclear compartment **(B)**. HeLa cells were infected at an MOI of 5 × 10^3^ with AAVrh.10-WT or S671A vectors. The sub-cellular localization of vectors as mean value of fluorescence in either the cytoplasm or nucleus was assessed as total area of fluorescence (pixel^2^) per visual field by Image J analysis software. ^*^*P* < 0.05.

Supporting Information Figure S3 Ubiquitination profile of AAVrh.10-S671A mutant is similar to WT-AAVh.10 vector. **(A)** WT-AAVrh.10, AAVrh.10-S671A, and AAVrh.10-K333R vectors (positive control mutant affecting ubiquitination) (3 × 10^8^ viral particles) were used to perform ubiquitin conjugation assay and further immunoblotting was performed to document the ubiquitination profile **(B)** Immunoblotting profile of AAV capsid proteins, VP1-3 which were used as a loading control.

Supporting Information Figure S4 AAV1-S669A mutant vectors demonstrate increased rate of intracellular trafficking when compared to AAV1 wild type vectors. Live cell imaging was carried out in HeLa cells infected with labeled AAV1 vectors at an MOI of 1 × 10^5^ using an Olympus confocal microscope. Live cell images of **(A)** WT-AAV1, **(B)** AAV1-S669A vectors were captured for 2.5 h.

Supporting Information Table S1 Nucleotide sequence of the primers used for site specific mutations in AAVrh.10 capsid (targeted codons have been underlined).

Supporting Information Table S2 Neutralizing antibody titers in animals that received AAVrh.10-WT or AAVrh.10-S671A vectors (*n* = 3).

Supporting Information Video 1 The video shows the live-cell imaging of viral trafficking in HeLa cells infected with WT-AAVrh.10 vectors at an MOI of 1 × 10^5^.

Supporting Information Video 2 The video shows the live-cell imaging of viral trafficking in HeLa cells infected with AAVrh.10-S671A vectors at an MOI of 1 × 10^5^.
