# Supplementary figures and images for: Optimized AAV rh.10 Vectors That Partially Evade Neutralizing Antibodies during Hepatic Gene Transfer
Source: Front Pharmacol. 2017 Jul 17;8:441. doi: 10.3389/fphar.2017.00441 (PMC5511854; doi:10.3389/fphar.2017.00441)

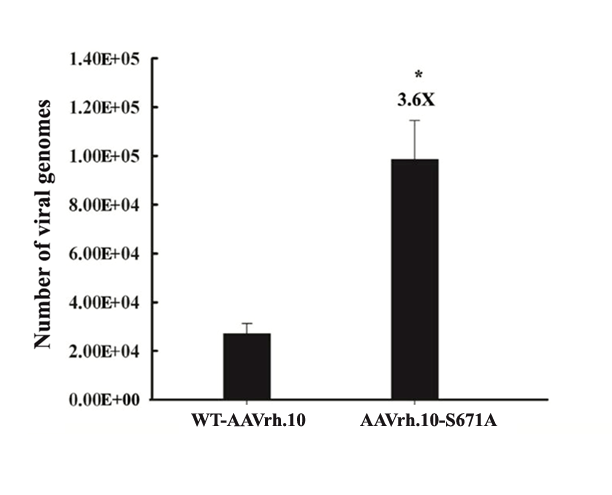

Supplement: Supporting Information Figure S1 — Cellular uptake is enhanced with modified AAVrh.10-S671A vectors. HeLa cells were infected at an MOI of 5 × 10^3^ with AAVrh.10-WT or S671A vectors. Genomic DNA was isolated 3 h post-infection and the vector copy number was measured by qPCR. The data was analyzed by student's t-test and the vector copy number in diploid genome are expressed as mean ± SD of two independent triplicate analysis. *p < 0.05 when compared to AAVrh.10-WT infected cells. [file Image1.TIF]

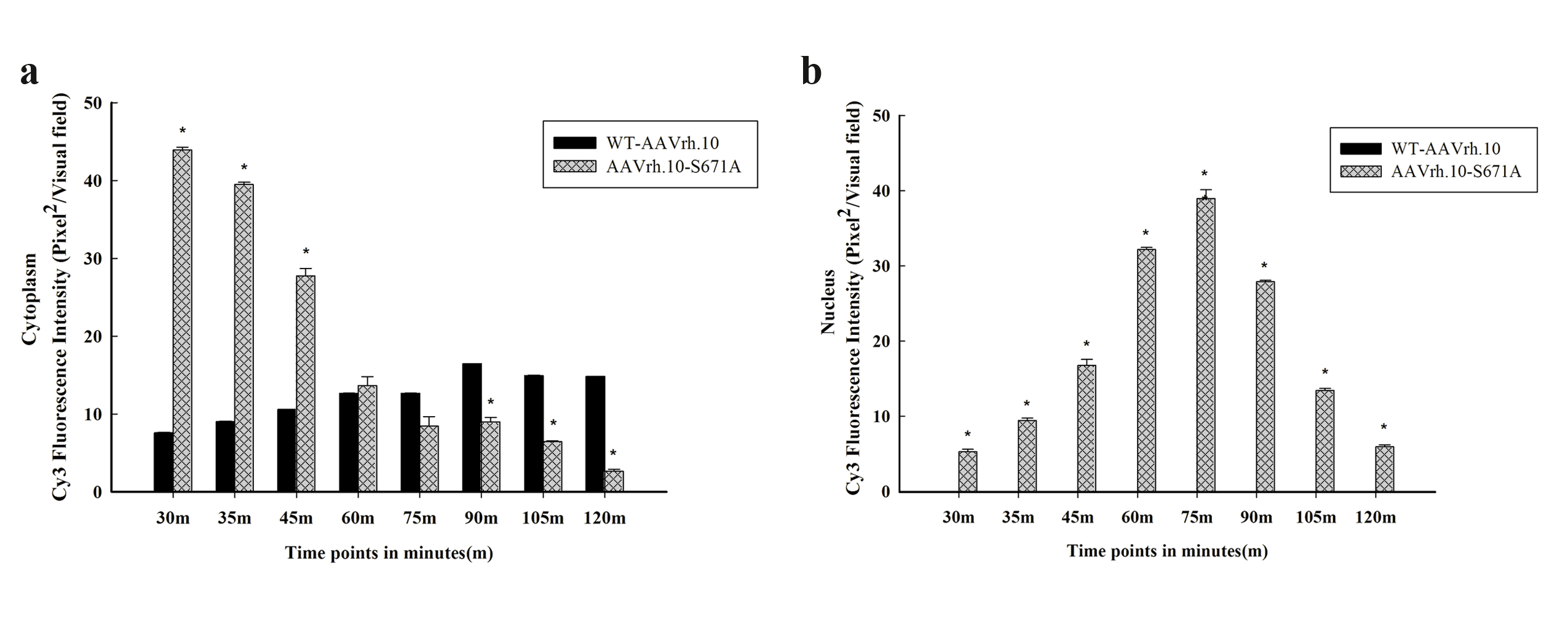

Supplement: Supporting Information Figure S2 — Quantitative estimation of viral trafficking in the cytoplasmic (A) or nuclear compartment (B). HeLa cells were infected at an MOI of 5 × 10^3^ with AAVrh.10-WT or S671A vectors. The sub-cellular localization of vectors as mean value of fluorescence in either the cytoplasm or nucleus was assessed as total area of fluorescence (pixel2) per visual field by Image J analysis software. *P < 0.05. [file Image2.TIF]

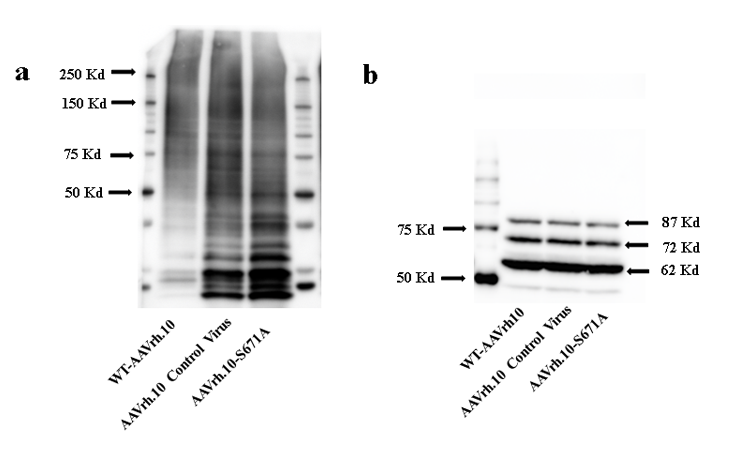

Supplement: Supporting Information Figure S3 — Ubiquitination profile of AAVrh.10-S671A mutant is similar to WT-AAVh.10 vector. (A) WT-AAVrh.10, AAVrh.10-S671A, and AAVrh.10-K333R vectors (positive control mutant affecting ubiquitination) (3 × 10^8^ viral particles) were used to perform ubiquitin conjugation assay and further immunoblotting was performed to document the ubiquitination profile (B) Immunoblotting profile of AAV capsid proteins, VP1-3 which were used as a loading control. [file Image3.TIF]

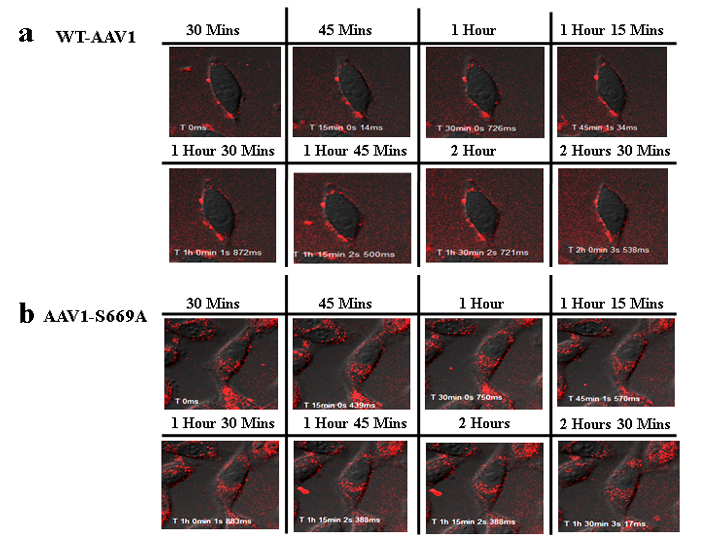

Supplement: Supporting Information Figure S4 — AAV1-S669A mutant vectors demonstrate increased rate of intracellular trafficking when compared to AAV1 wild type vectors. Live cell imaging was carried out in HeLa cells infected with labeled AAV1 vectors at an MOI of 1 × 10^5^ using an Olympus confocal microscope. Live cell images of (A) WT-AAV1, (B) AAV1-S669A vectors were captured for 2.5 h. [file Image4.TIF]
