# Supplementary material for: Optimized AAV rh.10 Vectors That Partially Evade Neutralizing Antibodies during Hepatic Gene Transfer
Source: Front Pharmacol. 2017 Jul 17;8:441. doi: 10.3389/fphar.2017.00441 (PMC5511854; doi:10.3389/fphar.2017.00441)
Supplement: Supporting Information Table S1 — Nucleotide sequence of the primers used for site specific mutations in AAVrh.10 capsid (targeted codons have been underlined). [file Tables1and2.DOCX]

**Supporting Information Table S1:** Nucleotide sequence of the primers used for site directed mutagenesis of AAVrh.10 capsid.

| **^RESIDUE^** | **^SEQUENCE ( 5’ to 3’)^** | **^NUCLEOTIDE CHANGE^** |
| --- | --- | --- |
| ^AAVrh10 S492A^ | ^Wild Type Primer Sequence:-^  ^gcagcaacgcgtc^**^Tcc^**^acgacactgtc^  ^Mutant Primer Sequence:-^  ^gcagcaacgcgtc^**^gcc^**^acgacactgtc^ | **^TCC🡪GCC^** |
| ^AAVrh10 S501A^ | ^Wild Type Primer Sequence:-^  ^ctgtcgcaaaataacaac^**^AGc^**^aactttgcctggaccgg^  ^Mutant Primer Sequence:-^  ^ctgtcgcaaaataacaac^**^gcc^**^aactttgcctggaccgg^ | **^AGC🡪GCC^** |
| ^AAVrh10 S671A^ | ^Wild Type Primer Sequence:-^  ^caagctaagctggcg^**^Tcg^**^ttcatcacgcagt^  ^Mutant Primer Sequence:-^  ^caagctaagctggcg^**^gcg^**^ttcatcacgcagt^ | **^TCG🡪GCG^** |
| ^AAVrh10 T108A^ | ^Wild Type Primer Sequence:-^  ^gcgtctgcaagaagat^**^Acg^**^tcttttgggggc^  ^Mutant Primer Sequence:-^  ^gcgtctgcaagaagat^**^gcg^**^tcttttgggggc^ | **^ACG🡪GCG^** |
| ^AAVrh10 T252A^ | ^Wild Type Primer Sequence:-^  ^ctgggccctcccc^**^Acc^**^tacaacaacca^  ^Mutant Primer Sequence:-^  ^ctgggccctcccc^**^gcc^**^tacaacaacca^ | **^ACC🡪GCC^** |
| ^AAVrh10 T674A^ | ^Wild Type Primer Sequence:-^  ^tggcgtcgttcatc^**^Acg^**^cagtacagcacc^  ^Mutant Primer Sequence:-^  ^tggcgtcgttcatc^**^gcg^**^cagtacagcacc^ | **^ACG🡪GCG^** |
| ^AAVrh10^  ^K84R^ | ^Wild Type Primer Sequence:-^  ^gcctacgaccagcagctc^**^aaa^**^gcgggtgac^  ^Mutant Primer Sequence:-^  ^gcctacgaccagcagctc^**^aga^**^gcgggtgac^ | **^AAA🡪AGA^** |
| ^AAVrh10^  ^K137R^ | ^Wild Type Primer Sequence:-^  ^tggttgaggaaggcgct^**^aAg^**^acggctcct^  ^Mutant Primer Sequence:-^  ^tggttgaggaaggcgct^**^agg^**^acggctcct^ | **^AAG🡪AGG^** |
| ^AAVrh10^  ^K333R^ | ^Wild Type Primer Sequence:-^  ^gcagaatgaaggcacc^**^aAg^**^accatcgccaataacc^  ^Mutant Primer Sequence:-^  ^gcagaatgaaggcacc^**^agg^**^accatcgccaataacc^ | **^AAG🡪AGG^** |

**Supporting Information Table S2:** Neutralization antibody titre values in serum of animals adminstered with either AAVrh.10-WT and AAVrh.10-S671A vectors.

| **Sample** | **Nab50 Titre** |
| --- | --- |
| AAVrh.10WT | 320 |
| AAVrh.10-S671A | 160 |
